# A semi-automated methodology for finding lipid-related GO terms

**DOI:** 10.1093/database/bau089

**Published:** 2014-09-10

**Authors:** Mengyuan Fan, Hong Sang Low, Markus R. Wenk, Limsoon Wong

**Affiliations:** ^1^Department of Computer Science, National University of Singapore, Singapore 117417, ^2^NUS Graduate School of Integrative Science and Engineering, National University of Singapore, Singapore 117456, ^3^National Research Foundation, Singapore 138602, ^4^Department of Biochemistry, National University of Singapore, Singapore 117599 and ^5^Department of Pathology, National University of Singapore, Singapore 119074

## Abstract

**Motivation:** Although semantic similarity in Gene Ontology (GO) and other approaches may be used to find similar GO terms, there is yet a method to systematically find a class of GO terms sharing a common property with high accuracy (e.g. involving human curation).

**Results**: We have developed a methodology to address this issue and applied it to identify lipid-related GO terms, owing to the important and varied roles of lipids in many biological processes. Our methodology finds lipid-related GO terms in a semi-automated manner, requiring only moderate manual curation. We first obtain a list of lipid-related gold-standard GO terms by keyword search and manual curation. Then, based on the hypothesis that co-annotated GO terms share similar properties, we develop a machine learning method that expands the list of lipid-related terms from the gold standard. Those terms predicted most likely to be lipid related are examined by a human curator following specific curation rules to confirm the class labels. The structure of GO is also exploited to help reduce the curation effort. The prediction and curation cycle is repeated until no further lipid-related term is found. Our approach has covered a high proportion, if not all, of lipid-related terms with relatively high efficiency.

**Database URL:** http://compbio.ddns.comp.nus.edu.sg/∼lipidgo

## Introduction

Gene Ontology (GO) (1) is the most widely used controlled vocabulary that provides functional annotation for gene product. GO contains three orthogonal sub-ontologies—viz., cellular component (CC), molecular function (MF) and biological process (BP). Each sub-ontology covers different aspect of biology and is represented as a rooted, directed acyclic graph with multiple inheritance property in which a more specific child term may have more than one parent, which is more general. For example, the term GO:0005739 ‘mitochondrion’ has two parents GO:0043231 ‘intracellular membrane-bounded organelle’ and GO:0044444 ‘cytoplasmic part’. There are several types of relationships between parent and child node in GO: ‘is-a’, ‘part-of’, ‘regulates’, etc (2). Only ‘is-a’ relationship is considered in our project because our interest is to find a class of similar GO terms—e.g. GO terms with lipid-relatedness property—and only in ‘is-a’ relationship the child term inherits the property or attribute of the parent. In fact, the ‘is-a’ relationship is transitive. When a parent term is lipid related, all its child terms are also lipid related, and the child terms of all the child terms are also lipid related and so on. The logical consequence of this is the ‘inheritance constraint’ property: if an ancestor term is lipid related, then all its descendant terms are necessarily lipid related. However, the inverse of above statement may not necessarily hold true: when an ancestor/parent term is not lipid related, the descendant/child term may still be lipid related. For example, GO:0006644 ‘phospholipid metabolic process’ is lipid related but its parent term GO:0006796 ‘phosphate-containing compound metabolic process’ is not necessarily lipid related. Another property of GO is the ‘true-path’ rule: genes annotated by more specialized child terms are transitively and implicitly annotated by the more general parent terms as well, i.e. a gene annotated by the term ‘mitochondrion’ is, transitively and implicitly, also annotated by the term ‘cell part’ (via intermediate terms ‘cytoplasmic part’ and ‘intracellular part’).

There have been a number of studies on finding related GO terms. A popular concept for comparing the similarity between a pair of GO terms is semantic similarity, consisting of three main classes: node-based approach, edge-based approach and hybrid approach (3). Central to the node-based approach is information content (IC), which gives a measure of how specific and informative a term is. In the case of GO, the frequency p of a term is the ratio of the number of genes annotated by that particular term to all GO annotations. The smaller the frequency, the more informative that term is, and this relationship is mathematically represented as IC = −log(p). Resnik (4) proposed a simple measure of semantic similarity between any two nodes in a directed acyclic graph: the IC of their lowest common ancestor (LCA), and if there are multiple LCA, the one with the highest IC, is used. This measure was later adapted for GO and achieved good results in some independent assessment studies despite its simplicity (5). There are variations of this formula for calculating semantic similarity: (6–8). There are also edge-based methods (9) and hybrid methods using both node and edge (10).

All these methods involving semantic similarity inherently depend on how close the two GO terms are in the GO structure. However, for the purpose of finding a class of similar GO terms, we may need to look in different parts of the GO tree. For example, both GO:0006631 ‘fatty acid metabolic process’ and GO:0034378 ‘chylomicron assembly’ are lipid related but the only common ancestor besides the root is ‘single-organism process’, which is a general term. To achieve that purpose, other data sources are needed, such as co-annotation to the same proteins or genes and co-occurrence in literature. Jin and Lu (11) adopt a text-mining approach and construct a word-usage profile as a feature for each GO term by collecting the words from PubMed records (titles and abstracts) based on the PMIDs associated with that GO term to reflect the semantic context of the literature associated with that GO term. Riensche’s XOA (Cross-Ontological Analytics) (12) uses co-annotation data by constructing a matrix having GO terms as row and gene products as columns, with the value of each cell of the matrix being a count of association between the corresponding GO term and gene product. This framework is able to calculate semantic similarity of terms from different sub-ontologies, which is not found in other methods above but is highly useful for finding a class of GO terms, as they may span all three sub-ontologies.

Unlike others who focus on pair-wise similarity, the aim of our work is to find a class of GO terms sharing a certain property. In particular, we develop an approach to identify the class of lipid-related GO terms. We choose the class of lipid-related GO terms because lipids and their metabolites are very important and play an essential role in many BPs relating to energy homeostasis, signaling, neurobiology, infectious diseases and so on. Moreover, one of the most important goals in the emerging field of lipidomics is identification of genes and proteins involved in lipid metabolism and other relevant processes (13). Like biologists in other subfields, lipid researchers are interested in elucidating lipid metabolism functions of gene products, and most of them use GO for functional annotation. Therefore, the identification of lipid-related GO terms will help lipid research, either directly or in combination with other bioinformatics tools involving GO terms. For example, lipid-related GO terms were used to identify novel lipid-associated complexes involved in liver cancer progression (14).

A GO term is lipid related if lipids or lipid complexes (liposaccharides, lipoprotein, etc.) are involved in the term as reactants, participants or structural components. To be more exact, we have made a set of specific curation rules in Section 2.5 to clearly define what terms are lipid related. As we are only interested in binary classification, the parents or ancestors of lipid-related term, which are not necessarily lipid related, can be in the category of non– lipid-related terms.

For some terms it is easy to recognize that they are lipid related, while some other terms may associate with lipid function in an obscure way. A lipid-related GO term is defined as explicitly lipid related if there is lipid-related keyword (KW) in its term name or term definition. The rest of the lipid-related GO terms are implicitly lipid related, and detailed knowledge about the term is required for recognition of the association of lipid function to the term.

Searching lipid-related GO terms comprehensively across all three sub-ontologies of GO is a nontrivial task. While some terms are explicitly lipid related, often lipid functions are implicitly associated with GO terms. Usually, highly specific knowledge about the relevant processes, pathways, etc. is needed to realize that these latter GO terms are related to lipids. Furthermore, human manual curation on all GO terms is time-consuming and a tedious task, especially for the BP sub-ontology. There are about 40,000 GO terms, covering many concepts in many subdisciplines of biology. And, for BP terms, it demands even more detailed knowledge of the curator as each BP term represents a series of events accomplished by one or more ordered assemblies of MFs.

Our approach combines computational prediction with manual curation to achieve high accuracy and high coverage at less curation effort. We adopt the following incremental expansion strategy. With the help of an expert curator, we first obtain a list of high-quality manually curated lipid-related terms as well as non–lipid-related terms. This list forms the initial gold standard that covers all terms in the MF and CC sub-ontologies and a small portion of the BP sub-ontology from a particular version of GO (June 2009). A GO term annotated to one set of gene products is likely to be similar to other GO terms annotated to the same set of gene products (5). Using this observation and the properties of GO structure, we develop a machine learning method that can be used to expand the list of lipid-related terms from the gold standard. Those terms with high prediction scores (i.e. likely to be lipid related) are then examined by a human curator following specific curation rules so that the predicted class labels (i.e. lipid related vs non-lipid related) can be confirmed. The procedure of prediction and curation is repeated until no further lipid-related terms are found. The rest of the terms, likely to be non-lipid related, are not manually examined.

In our previous work, we have built a Web site for users to view and download information on lipid-related GO terms (15). This article elaborates the methodology of finding lipid-related GO terms and addresses related issues such as dealing with multiple versions of GO and balancing curation effort and accuracy. In our project, the following notations are used to describe class labels of GO terms according to sub-ontology and lipid relatedness: BP+ for lipid-related BP terms, BP− for non–lipid-related BP terms, BP? for BP terms whose lipid relatedness has not been examined by a curator. Similar notations are defined for CC terms (CC+, CC−, CC?), MF terms (MF+, MF−, MF?) and GO terms in general (GO+, GO−, GO?). As a result of the inheritance constraint, all the children and descendants of GO+ are also GO+ (sharing the lipid-related property). Also, no GO− has GO+ parents or ancestors (contrapositive). However, the children or descendants of GO− can be either GO+ or GO−.

## Methods

### Collection of gold standard lipid-related GO terms

With the help of an expert curator, we obtained a list of high-quality, manually curated, lipid-related terms as well as non–lipid-related terms following predefined curation rules. Non–lipid-related terms are also provided by the curator to be used as negative controls in the classifier. This list forms the initial gold standard that covers all terms in the MF and CC sub-ontologies and a small portion of the BP sub-ontology from the June 2009 version of GO. The curation rules will be elaborated later in the curation section.

### Database construction

The relational database of GO was downloaded from the official Web site. Multiple versions of GO were used: 5 June 2009 for the gold standard and 8 April 2013 for the simulation experiment. As GO keeps evolving and we want to keep it up to date, multiple versions of GO are kept. Important tables in the original GO database include ‘term’, ‘term2term’ and ‘association’. To add class label of GO terms and other relevant information, we built our own version of the table ‘term’, naming it as ‘myterm’. As our interest is whether a GO term has the property of lipid relatedness, we kept only the is_a relationship in the table ‘term2term’ and built the table ‘myterm2term’. We also calculated the transitive closure of ‘myterm2term’ into a table ‘mypath’ to capture ancestor–descendant relationship. With the table ‘mypath’, we can check the inheritance constraint easily to ensure the integrity of the class label. Table ‘association’ is about which GO term is associated with which gene product. We processed the table ‘association’ into the table ‘myassociation’ by removing negative associations. GO associations from all available organisms are used, and associations with all evidence codes are kept including ‘Inferred from Electronic Annotation' (IEA).

### Mapping lipid-related GO terms between different GO versions

GO is frequently updated to meet the need of accumulation of biological knowledge. It is often necessary to map the list of lipid-related GO terms as well as non–lipidrelated controls between different GO versions. For example, our list of gold-standard GO terms is from an older version (June 2009), but we are also interested to label terms in the latest version of GO. Therefore, mapping is necessary, and several issues have to be addressed. First, we discard those gold-standard terms that become obsolete. We also re-examine whether the terms are lipid related if there are changes in term names or term definitions. Furthermore, the inheritance constraint may be potentially violated (e.g. a previous GO+ parent may now have GO−/GO? child) owing to some change in the GO structure or addition of new terms. We manually examine the substructures in GO where there is anomaly, and we resolve the violation in two ways: either the parent term is converted into GO− or the child term is converted into GO+. For example, the new term GO:0036042 ‘long-chain fatty acyl-CoA binding’ (as of April 2013 version of GO) became the child term of GO:0000062 ‘fatty-acyl-CoA binding’. We resolved the conflict by changing the class label of the child term GO:0036042 to GO+, as apparently it is lipid related.

### Feature generation

Features for identifying lipid-related GO terms were generated in two ways: by KWs and by co-annotation. KWs can be used to find explicitly lipid-related GO terms by looking at term name or term definition. A list of KWs containing names of common lipid-related compounds was picked by the expert curator in our lab when he was building the gold-standard set, and we extended the list into 36 KWs after consulting with the LIPID MAPS classification system (16). The 36 KWs are lipid, adipo, lipo, vesicle, lipase, fatty, fats, abscisic, eicosanoid, leukotriene, docosanoid, octadecanoid, arachid, cholesterol, sterol, steryl, steroid, glucocorticoid, bile acid, phosphatidyl, ceramide, acetylcholine, gluconate, prenol, terpene, isoprene, isoprenoid, terpenoid, geranyl, quinones, hopanoid, glycerophospho, sphingo, ganglioside, acyltrehalose and acylaminosugar.

For a particular KW feature, we encode it as 1 if the KW appears as a case-insensitive substring in its term name or term definition and 0 otherwise. Thus, the KW may appear as a separate word itself or within a composite word (e.g. lipo appearing in lipoprotein). The form of these KWs was carefully chosen to ensure good accuracy and coverage. For example, ‘lipid’ is chosen rather than ‘lipids’ because it offers a better coverage. On the other hand, ‘Fats’ is preferred over ‘fat’ as otherwise ‘fate’ will be counted as hit, which is not lipid related in general. Database search has shown that to have a lipid-related meaning, the majority of the substring ‘fat’ appears in two forms, ‘fats’ or ‘fatty’, and therefore, we keep both forms. Similarly, we keep both the noun form ‘sterol’ and the adjective form ‘steryl’. This step simplifies natural language processing procedure like dealing with plural forms, part of speech, etc.

On the other hand, GO terms annotated to the same sets of gene products are likely to be terms that describe highly related biological entities and processes. As gene product is the bridge between GO terms with known class label (including both GO+ and GO−) and those whose class label we want to predict (GO?), we first give a ‘lipid-relatedness weight’ to each gene product as follows. For each GO sub-ontology, a separate weight is designed because BP, CC and MF carry different aspects of information. For example, a gene product g may be explicitly annotated with 3 BP+, 2 BP−, 2 BP?, no CC+, 4 CC−, 2 CC?, no MF+, no MF− and 1 MF? terms. The BP weight of a gene product g, BP_weight(g), is number of BP+ divided by number of BP+ and BP− terms, which in this case is 3/(3 + 2) = 0.6. Similarly, CC_weight(g) is 0/(0 + 4) = 0. MF weight for g does not exist, as there is no MF with known class label (e.g. MF+ or MF−). During the calculation of gene product weight, we do not propagate annotations to ancestor terms, and thus, only explicit annotations are considered.

The next step is to calculate co-annotation feature scores for the term, which are based on lipid-relatedness weights of gene products annotated with that term. Here we consider gene products not only explicitly annotated with that term but also its descendant terms. For a term t, suppose there are four distinct gene products that are annotated with the term t or its descendant terms. Three of the four gene products have BP weights, which are 0.2, 0.3 and 0.7. The BP score for the term t, BP_score(t), is the sum of BP weights divided by the number of BP weights, which in this case is (0.2 + 0.3 + 0.7)/3 = 0.4. CC and MF scores are calculated in a similar fashion. By the nature of our formula, the sub-ontology scores (BP, CC and MF scores) take values between 0 and 1. For a term, if there is no associated gene product or none of the associated gene products has gene product weight for a sub-ontology, we set the corresponding sub-ontology score to zero.

Finally, we have three sub-ontology feature scores derived as described above from co-annotation in addition to the 36 KW features. These 39 features are used to build our classifier and make prediction on GO terms with unknown label. The classifier is a support vector machine (SVM) with linear kernel.

### Curation

The development of curation rules is not a simple process: the rules are being developed incrementally on multiple GO versions with a few curators involved. In the original gold standard, there were only four rules for CC and MF sub-ontology and no rules for BP. With more terms curated and more experiences accumulated, we modified and expanded the rules to make the set of curation rules as consistent and informative as possible. We set the scope of lipid by following the definition of lipids based on LIPID MAPS (16), which is a popular portal for the lipid community and has a comprehensive database containing lipid molecular structure and lipid proteome. Then we proceed from the scope of lipid to the scope of lipid-related GO terms, where extra clarification is needed and several curation rules are provided. Moreover, we have captured several common patterns of implicitly lipid-related GO terms and stated them in rule form. However, a perfect division of GO terms into lipid-related or non–lipid-related class is unrealistic. We will elaborate the issues of curation in the curation section of the discussion.

The original curation rules for the gold standard for the June 2009 version of GO are as follows:

- If a term is in the form of ‘X transporter activity’, we consider the term as lipid related if X is involved in a lipid-related process.
- In the case of catalytic activity or reaction, if any substrate or product is lipid related, the term is lipid related.
- Polyketides are not considered as lipids, while lipopolysacharides, glycolipids and lipoproteins are considered as lipid related.
- Transmembrane signaling receptors are considered lipid-related terms with the explicit mentioning of the KW ‘Transmembrane’.

The final curation rules (for GO version April 2013) are as follows:

1. A term is considered as lipid related if its term name or term definition contains explicit KWs related to lipid. Exceptions are the following:
  - Short-chain fatty acids (those fatty acids with a chain length of ≤5) and their CoA forms are not considered as lipid related in general (e.g. formic acid, acetic acid, propionic acid and acetyl-CoA).
  - Negation of KWs.
  - Presence of KW in a way that does not entail the participation of that KW in the term. (e.g. in a disjunctive phrase like ‘lipid or protein’.)
2. Polyketides are not considered as lipid related, while lipopolysacharides, glycolipids and lipoproteins are considered as lipid related.
3. In the case of catalytic activity or reaction, if any substrate or product is lipid related, we consider the term as lipid related.
4. ‘Membrane-bounded vesicle’ is considered as lipid related.
5. Regulation of lipid-related metabolic processes is lipid related.
6. Regulation of membrane potential is considered as lipid related in general.
7. A metabolic process involving X is considered as lipid related if X is commonly incorporated into lipids or lipid complexes, e.g. GO:0006114 ‘glycerol biosynthetic process’.
8. A BP term is considered as lipid related if that process involves a change to membrane or vesicle including folding, invagination, pinching off and fusion.
9. A BP term is considered as lipid related if that process involves modification, breakdown or other changes of lipids or lipid complexes.
10. We consider a term as lipid related if it can be linked to a lipid-related function in other ways. For example, GO:0002024 ‘diet-induced thermogenesis’ is lipid related, as it is closely related to fatty acid catabolism for heat generation.
11. Protein complexes in membrane part are not considered as lipid related in general. They are lipid related if they are involved in some metabolism of lipid or lipid complexes or play an essential role like membrane fusion, vesicle formation and so on. Those CC parts that are intrinsic to membrane are also considered as lipid related.

### Iterative prediction

Machine learning algorithms are generally well behaved in the sense that, when we have a larger training set, the resulting classifier can be expected to be more accurate. Thus, instead of building our classifier only once and making all the predictions followed by curation, we do it in an iterative manner that hopefully leads to less curation effort being wasted on false-positive (FP) results. The overall iterative procedure is like this: starting with an initial list of GO terms with known class labels, we generate features, build a classifier and make predictions on all terms whose class label is unknown. Then we select a batch of top-ranked terms with the highest prediction scores (i.e. terms most likely to be lipid related) as candidates for curation. Terms in the batch are manually curated following the curation rules defined in the earlier section, and their class labels are updated. We also apply a postprocessing step to enforce the inheritance constraint: descendant terms of newly curated GO+ become GO+; ancestor terms of newly curated GO− become GO−. Now there is a larger set of GO terms with known class labels that can be used as training instances, and the whole procedure is repeated to identify more lipid-related GO terms.

### Assessing the iterative prediction procedure

We use a fraction of our final list (see Results section) of curated GO+ and GO− terms to run simulated experiments, and see how our methodology recovers the rest of the GO+ terms in our final list of curated GO+ and GO− terms. As we do not manually curate all GO terms, those terms with unknown class label (GO?) are excluded in the evaluation of curation efficiency.

To test our hypothesis that iterative prediction saves curation effort compared with doing just one round of curation, we perform experiments with six different starting conditions, some with more GO terms used as the initial training instances and some with less. The first starting condition is nonrandom: we manually picked six representative GO terms, one positive and one negative control for each sub-ontology. They are general terms with a relatively large number of descendants. We apply the inheritance constraint to the three GO+ terms to include their GO+ descendants into the training set. The three GO− terms were specially chosen such that all the descendants of the three GO− terms are also GO− (this is not true in general); by the inheritance constraint, the ancestors of the three GO− and their GO− descendants are also GO−, and all of them are included in the training set as negative control. The representative GO terms, their class labels and so on are summarized in Table 1. For starting conditions two to six, we randomly select x% of our final list of curated GO+ and y% of our final list of curated GO− as the initial training instances. We apply the inheritance constraint: the descendants of GO+ terms in the training set are also added to the training set as GO+ terms; the ancestors of GO− terms in the training are also added to the training set as GO− terms. Thus, the actual proportion of terms with known class label is higher. The proportions are given in the first two rows in Table 2 for the positive and negative control, respectively. The number on the left side of the slash is the proportion intended for training before applying the inheritance constraint, and the number to the right of the slash is the proportion after applying the inheritance constraint, which is also the actual proportion of training instances used in the experiment. The rest of the terms are used as test set with the number for different starting conditions shown in the last two rows of Table 2.

**Table 1.** Starting condition #1, nonrandom

GO accession number	Class label	Term name	Number of terms in the subtree rooted at the term
GO:0006644	BP+	Phospholipid metabolic process	105
GO:0016020	CC+	Membrane	133
GO:0008289	MF+	Lipid binding	70
GO:0006767	BP−	Water-soluble vitamin metabolic process	44 (178^a^)
GO:0030880	CC−	RNA polymerase complex	17 (31^a^)
GO:0000496	MF−	Base pairing	85 (96^a^)

^a^Number of terms by including their GO− ancestors by the inheritance constraint.

**Table 2.** All six starting conditions for iterative prediction

Starting condition	#1	#2	#3	#4	#5	#6
% GO+ in the training set	6.5	2/6.7	5/12.6	20/45.3	50/76.1	80/92.3
% GO− in the training set	2.1	5/15	10/22.9	20/36.6	50/65.3	80/86.8
Number of GO+ in the test set	4366	4386	4061	2758	1145	358
Number of GO+ and GO− in the test set	18,305	16,529	15,047	11,814	6162	2210

In each iteration, prediction is made on the test set, and a ranked list is generated based on prediction scores, with higher score corresponding to being more likely lipid related. We select a batch of only a fixed number of terms from the top of the list with the highest prediction scores. Those terms in the batch with a negative prediction score are discarded, and the rest is checked against their real class labels in the final list, which is assumed here to be 100% correct even though it is not perfect. In the case that the real class label is GO+, it is counted as a true positive (TP); if the real class label is GO−, it is counted as an FP; if the real class label is GO?, we just ignore the term. This checking of real-class labels in the experiment simulates the manual curation for *de novo* discovery of lipid-related GO terms using the methodology in this article. As a bonus, without additional curation effort, we apply the inheritance constraint to get descendant terms of those TP in the batch as lipid related (denoted as desc+) and ancestor terms of FP in the batch as non-lipid related (denoted as ance−). Terms from TP, FP, desc+, ance− and their newly obtained class labels will be used as training instances in the subsequent iterations. They together with the ignored terms (those with class label GO?) are excluded from test sets in the subsequent iterations. As a result of changing training and test sets, each subsequent iteration has a new starting condition, but the procedure remains the same. The number of terms in the batch is set to be relatively smaller first and increases in later iterations. The rationale for this is that in the first few iterations, there are fewer training instances and even an addition of 100 terms can make a significant impact on the classifier but, at later iterations, the impact is not that great when most of GO+ have already been discovered in earlier iterations. Therefore, we set 100 terms per batch in the first five iteration; 200 for iterations 6–10; 300 for iterations 11–15; and 500 for iterations 16–25. Although the number of terms per batch seems to be large, especially for later iterations, we deliberately set it to be so because the test set may contain terms with GO? class label, which are ignored in the evaluation and cannot affect future prediction. We still want to keep those GO? terms in the test set, as these additional terms may provide us insight into our prediction procedure and curation rules.

For performance comparison purpose, we also ran the experiments using a non-iterative version with the same six starting conditions. The classifiers are built, and the test sets are predicted and sorted into a single ranked list. We take the top 300 terms from this list with the highest prediction scores, and check their real class labels to get TP, FP and ignored GO? terms as we did in the iterative case. The inheritance constraint is applied to get desc+ and ance−. Terms from TP, FP, GO?, desc+, ance− are taken out from the single ranked list. Without training the classifier again, we repeat the checking procedure by taking the top 300 terms with the highest score from the reduced list, checking the class label, applying the inheritance constraint and reducing the ranked list for 20 times.

Another experiment was carried out to see the effect of different combination of feature scores on the performance. Instead of using all 39 features, we ran an additional set of experiments with only the 36 KW features. As before, both iterative and non-iterative versions are used and starting condition #1 and #2 were chosen for nonrandom and random case.

To evaluate curation efficiency, two numbers are needed. The first is curation effort or number of terms manually curated, which corresponds to number of terms in the TP and FP in our experiment, as they are the terms that the curator examines. The second is curation hit, or the number of lipid-related GO terms discovered, which includes not only TP found by curation but also the number of terms in desc+ derived from the inheritance constraint. These two numbers are cumulative with respect to iterations or batches. For example, the curation effort up to iteration #3 is the sum of the number of terms curated from iteration 1 to 3. Finally, the curation effort is plotted against curation hit as a measurement of curation efficiency. If the ratio of curation hit to curation effort is 1 or above, the effort is really worth it, as every term curated gives you at least one GO+ term on average.

$$\begin{array}{l} \text{Curation effort}=\text{TP}+\text{FP}\\ \text{Curation Hit}=\text{TP}+\left(\text{desc}+\right) \end{array}$$

Recall and precision are also given as alternative evaluation measure. Formulas are given below, where P is the total number of GO+ in the test set; since desc+ is derived from TP by inheritance constraint, the sum of it and TP is considered as ‘total true positive’ and thus appears accordingly in the formula. Similar to curation efficiency measure, the numbers in the formula are cumulative with respect to iterations or batches. Unlike the curation effort and hit evaluation measure, we keep those terms with the negative prediction score when selecting a batch of a fixed number of terms with highest prediction scores in each iteration. For more detailed result, please go to the supplementary material.

$$\begin{array}{l} \text{Recall}=\left(\text{TP}+\left(\text{desc}+\right)\right)/\text{P}\\ \text{Precision}=\left(\text{TP}+\left(\text{desc}+\right)\right)/\left(\text{TP}+\left(\text{desc}+\right)+\text{FP}\right) \end{array}$$

## Results

The number of GO terms during different stages of work is given in the Table 3, decomposed by sub-ontologies and lipid relatedness. We had a number of gold-standard GO terms to start with, covering all CC terms and MF terms and a small portion of BP terms in that version of GO. The gold standard even includes some terms that are associated with lipid function implicitly. The curation used for the gold standard then was not developed yet: it had only four rules, as specified earlier. As we are interested in the latest version of GO, we mapped the class label from the gold standard to the April 2013 version of GO. Some terms became obsolete and new terms were added, with the overall number of terms increase from 27,734 to 37,462. Because of changes in GO structure, we found many pairs of terms that violated the inheritance constraints. Moreover, a new set of curation rules is needed because the newer version of GO becomes more complicated, and there are many changes in term definitions, structures and so on (evolution and refinement of curation rule will be elaborated in the discussion). We resolved the violated inheritance constraints with new curation rules, and the distribution of class labels changed dramatically. The increase of BP+ was mainly due to the addition of new terms as descendants of gold standard BP+, and the decrease of CC+ and MF+ was largely caused by modification of curation rules. After that, we applied our iterative approach to find the rest of lipid-related GO terms in all three sub-ontologies, and the final results are given in the last column. So far, for the April 2013 version of GO, we manually curated 18,941 terms, comprising 4712 lipid-related GO term (GO+), 14,229 non–lipid-related GO terms (GO−). The rest of 18,521 terms, predicted not likely to be lipid related and not manually examined, are given the unknown class label (GO?).

**Table 3.** Number of GO terms

Class label	Gold standard with original curation rules (GO version: June 2009)	Gold standard mapped to April 2013 version of GO with original curation rules (may violate inheritance constraint)	After resolving inheritance constraints and with final set of curation rules (GO version: April 2013)	Final version (expanded with iterative prediction) with final curation rules (GO version: April 2013)
BP+	1639	1606	2225	2405
BP−	2309	2291	2650	5331
BP?	12,783	20,855	19,877	17,016
CC+	924	912	712	748
CC−	1461	1429	1845	1870
CC?	0	817	601	540
MF+	1736	1668	1495	1559
MF−	6882	6658	6956	7028
MF?	0	1206	1101	965
GO+	4299	4186	4432	4712
GO−	10,652	10,378	11,451	14,229
GO?	12,783	22,898	21,579	18,521
All	27,734	37,462	37,462	37,462

We first would like to know how good predictors of lipid-relatedness KW features are in general. Among the manually curated 18,941 terms mentioned in the previous paragraph whose class label are known, we decomposed them according to their class label and whether they have at least one KW (Table 4). The proportion of the terms with more than at least one KWs is only 13%, and it is expected to be even lower if the scope is over all GO terms, which include terms with unknown class label (GO?). Among those non–lipid-related GO terms, the great majority do not contain any KWs at all, which shows our chosen KWs are specific. On the other hand, less than half of the lipid-related terms contain any KWs, giving only a moderate sensitivity. In Table 5, we decompose the same 18,941 terms a bit differently: instead of splitting them into those having at least one KW and those not, we check whether a term or one of its ancestor terms contain at least one of the KWs and partition the set accordingly. Table 5 shows the number of terms according to this new way of partitioning: we see a moderate increased sensitivity but a great reduction in specificity, which does not seem to be a desirable trade-off. Thus, we do not propagate the KW features up to ancestors when we compute the KW feature score. Still, the sensitivity does not seem to be good enough. That is why sub-ontology scores are also used, especially for finding implicitly lipid-related terms.

**Table 4.** KWs and lipid relatedness

Number of terms	No KWs (87%)	Containing at least one KW (13%)
Non–lipid-related	14,014	224
Lipid-related	2406	2306

**Table 5.** KWs and lipid relatedness considering ancestors

Number of terms	Terms containing no KWs, not even any of its ancestors (67%)	Term itself or one of its ancestor contain at least one KW (33%)
Non–lipid-related	12,108	2121
Lipid-related	1204	3508

**Figure 1.**
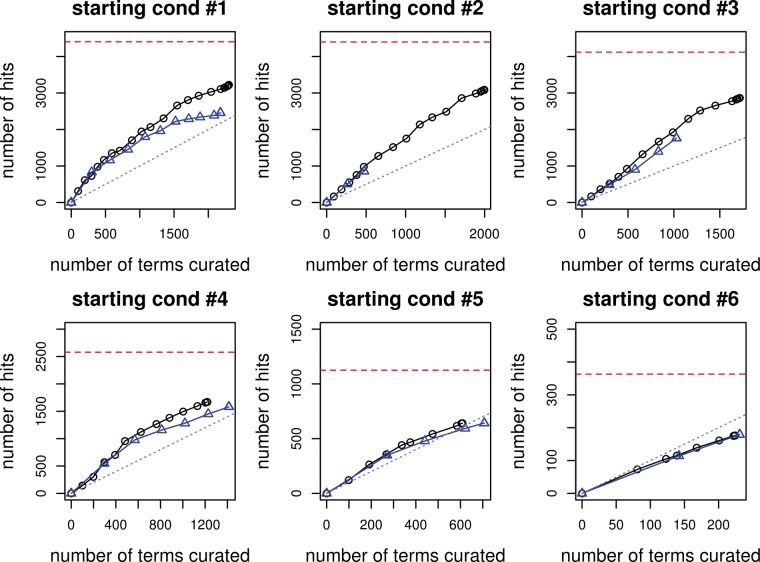
Curation efficiency for the six starting conditions. The black line with circle represents the iterative version, whereas the blue line with triangle represents the non-iterative one.

The curation efficiency curves are plotted in Figure 1 with the curation effort as the *x* axis and curation hit as the *y* axis. The horizontal red dotted line corresponds to total number GO+ terms in the test set, which sets the limit on the number of GO+ terms that can be found. The six subfigures correspond to six starting conditions, with the top left being the first one, top middle being the second one and so on. The black curve represents the performance of the iterative version, with the little circle on it corresponding to the number of iterations. The blue curve represents the performance of the non-iterative version, and the little triangle on the curve corresponds to the number of batches. The straight gray line is the break-even line, where curation effort equals to curation hits. Above the break-even line, on average one curation gives more than one discovery of lipid-related GO terms, and the curation effort is really worth it.

For all six starting conditions, the iterative version outperforms the non-iterative, and the difference is largest when the starting condition has the least number of terms used in training (i.e. starting condition #1, #2), and can be as many as 1000 terms. The difference between the two versions becomes smaller when a larger amount of training data is used, and by the time >90% are used as training data (starting condition #6), the two become almost the same.

Overall, as we curate more terms, there is a diminishing return. In the beginning, the number of GO+ terms found can be even greater than the number of terms predicted for curation, thanks to the inheritance constraint. As easier terms are found, much more curation effort is needed for recovering the same amount of GO+ terms, as reflected by the curve turning flat. With a reasonable amount of curation effort, >75% of the GO+ terms can be recovered from the test set. For instance, in the simulation experiment on the iterative version with starting condition #3, when ∼2500 terms are curated, 3000 of 4000 GO+ terms in the test set have been found. In this case, the semi-automatic approach seems to be a much more viable option than manually curating all the 14,451 terms (see Table 2 for statistics).

**Figure 2.**
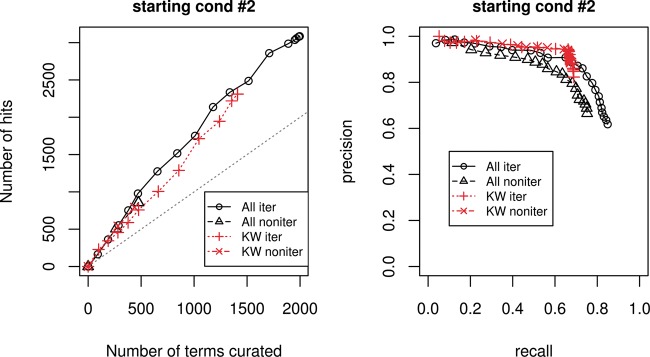
Evaluation measure for starting condition 2. Left graph corresponds to curation efficiency, right graph recall and precision.

Figure 2 shows evaluation measure for starting condition 2. The left graph is curation efficiency, and the right one is recall and precision. For both graph, the black curves represent using all features, red curves represent using only KW features; the solid lines correspond to the iterative cases and dotted lines correspond to the non- iterative cases. The straight gray line in the left graph is the break-even line, where curation effort equals to curation hits.

Overall, using only KW features performs slightly worse than using all features and at some points even gives more hits for the same amount of terms curated. However, using all features gives more terms with positive prediction scores, discovering more lipid-related terms. Overall, the curation efficiency is better using all features than using KW features alone.

As for the precision and recall graph, using KWs feature alone gives a slightly better precision at lower recall range. The precision for using only KWs feature drops abruptly when recall is after 0.7 where there are no more terms with KW features to be found. Using all features gives not only gives better maximum recall, but also better precision at higher recall range as it is able to handle ‘difficulty-to-find’ terms, or terms implicitly associated with lipid-related function.

Detailed result can be found in the Supplementary Materials. For both evaluation measures, the iterative approach outperforms the non-iterative one. We also tried other starting conditions, and the results were similar.

In the iterative approach, even after many iterations, there are some lipid-related GO terms in the test set yet to be found. For example, for starting condition #1, after the 25th iteration, 1182 GO+ terms are left among 13,195 terms with known class labels that have never been manually curated because of their low prediction score. These leftover 13,195 terms always appear from iteration 1 through 25 as test set and have a prediction score. For each iteration, the 13,195 terms are sorted from highest prediction score (most likely to be lipid related) to the lowest based on the prediction scores during that iteration. We are interested in the relative positions of 1182 GO+ terms within the sorted list of 13,195 terms while ignoring other terms and how these positions change with different iterations. As the classifier is expected to be more accurate with more training sets in later iterations than earlier ones, we hypothesize that the average rank of the 1182 GO+ terms improves with increasing iteration number among the sorted list of 13,195 terms, where the rank of a term inside this list is defined as follows: 1 if it has highest score, 2 second highest and so on. Thus, we test our hypothesis using staring condition #1 with four time point—viz., before iteration 1 and after iteration 8, 16 and 25—and perform one-sided Wilcoxon signed-rank test on their average rank.

The average ranks of the 1182 GO+ terms for the four points are 6469, 6232, 5573 and 5797. Even though the absolute difference is not huge, it is nonetheless statistically significant across some time points as given by the *P*-values in the Table 6. Overall, the rank is getting higher (nearer the top or smaller in the rank number). Our hypothesis is thus validated. Its significance is that, if further curation is to be done, iterative prediction helps find more TP results and saves curation effort.

**Table 6.** Paired *t*-test on ranks of lipid-related terms undiscovered after iteration 25

	After iteration 8	After iteration 16	After iteration 25
Before iteration 1	0.304	1.25E-04	1.15E-08
After iteration 8	–	4.97E-10	1.60E-05
After iteration 16	–	–	1.00E+00

## Discussion

### Difference between simulated experiments and our real discovery process

The simulated experiments with six different starting conditions given above are used mainly for illustrative purpose for readers to understand our methodology and get an idea of how much curation efficiency is improved by it. In our process of discovery of lipid-related GO terms, there are several complications. First, as our gold standard is from an older version of GO, we did GO version mapping and had to resolve inheritance constraint violations. Second, our curation rules, instead of being fixed, are being constantly refined as we accumulate experiences and adapted with new versions of GO; more details about this will be given in the next subsection. Thirdly, in our case, our number of gold-standard terms is more than enough, and it differs from the final version only by a few hundred additional terms that are either implicitly lipid related or from newly introduced GO terms if we do not take into account of our change in curation rules. In the end, 18,941 GO terms (or 50%) have been manually curated, of a total of 37,462 terms for the latest version of GO that we have worked with. In fact, we could save curation effort by having a much smaller set of gold standard, which proves to be successful in terms of curation efficiency based on our simulated experiment. Even a careful choice of six GO terms is good to start with, as shown in starting condition #1.

A caution is that during the real iterative prediction and curation process, while applying the inheritance constraint, it is always advisable to examine the descendant terms of TP terms (which is just decided by curator) to ensure that they are indeed GO+ instead of simply assuming this holds true, even though most of the time it is so. If at least one descendant term of, say, term X happens to be GO−, the curation decision need to be flipped and X becomes GO−. Fortunately, this additional procedure does not add much additional burden for curation, as in the most cases a cursory check is sufficient.

### Methodology

To exhaustively find all GO+ terms with only a small proportion of all GO terms curated is unrealistic. There is always a trade-off between curation effort and the number of GO+ terms recovered. As most GO+ terms have been recovered, the rest are becoming increasingly difficult to find. These ‘difficult terms’ are usually implicitly lipid related with few gene products assigned to them. So their lipid-relatedness property is not properly reflected in their KW feature scores and sub-ontology feature scores. However, this can be alleviated by serendipity of an expert curator: the discovery of one lipid-related term may remind him of another similar term if some association can be established based on his expert knowledge. For example, lipid plays many essential roles in neural process, the term GO:0043217 ‘myelin maintenance’ may remind the curator to search for more GO terms using KWs like ‘synaptic’, which are later filtered, examined and curated. This is especially helpful for finding implicitly lipid-related terms.

We are interested in independent evaluation of the quality of our list of lipid-related terms. There is a recent work GOplus (17) that provide explicit relationship between GO terms and ChEBI terms. With GO-ChEBI association and ChEBI ontologies, it is possible to use ChEBI term of interest to find our related GO term. As we are interested only in lipid-related GO terms, we made a query to find those GO terms that are associated to the descendants ChEBI terms of CHEBI:18059 ‘lipid’. The query returned 772 unique GO terms, among which 740 are in our final list of lipid-related GO term (April 2013). Their class label distribution is as follows: 584 BP+, 27 BP−, 15 BP?, 107 MF+, 6 MF− and 1 MF? (the details of the list of the terms are in the Supplementary Material). There are a few observations after examining those terms: the majority of them are lipid related; those terms with BP− and MF− class label are mostly short-chain lipids, which are not considered as lipid in our curation rule; some of the 15 BP? and 1 MF? are indeed lipid-related terms we miss, and they belong to the category of difficult-to-find term mentioned in the previous paragraph. Because most of the ChEBI terms related to lipids are descendant terms of CHEBI:18059 ‘lipid’, including fat, phospholipid, steroid, etc., we did not use other ChEBI term to find lipid-related GO terms. Compared with using ChEBI to find lipid-related term, our final list is much more comprehensive: contain 4712 lipid-related GO terms and also include CC sub-ontology.

To get an estimate of how many lipid-related GO terms are left undiscovered, we randomly sampled 200 GO terms with unknown class labels followed by manual curation. None were found to be lipid related. Our huge effort in curation gives an almost exhaustive list of lipid-related GO terms.

A few examples of FP results and false-negative (FN) results will be shown to explain why the classifier fails below. The first reason for FP is due to curation rules. Curation rule 1 considers GO:0046459 ‘short-chain fatty acid metabolic process’ as GO−, and by inheritance constraint its ancestor term GO:0006629 ‘lipid metabolic process’ are GO− even though it has high prediction scores. Another reason for FP is the negation of KW(s). For example, for term GO:0043264 ‘extracellular non-membrane-bounded organelle’ with term definition ‘organized structure of distinctive morphology and function, not bounded by a lipid bilayer membrane and occurring outside the cell’, the appearance of KW ‘lipid’ makes the prediction score high even though it means absence of lipid. The third reason for FP is inflated sub-ontology feature score by multi-functionality of the enzyme. The term GO:0047718 ‘indanol dehydrogenase activity’ means a function that catalyzes the non-lipid compound indanone into another non-lipid compound indanol, and the term is GO−. However, a majority of gene products annotated to this GO also catalyze reaction involving lipid compounds. For instance, human gene AKR1C3 corresponds to an enzyme that catalyzes retinal into retinol, and both compounds are closely related to vitamin A, which is a lipid. This enzyme also catalyzes some steroids. Thus, the sub-ontology scores, especially the MF score, are inflated and GO:0047718 becomes an FP. On the other hand, a majority of FNs are mainly owing to the lack of KW and has no gene products association or associated with gene products that are not assigned to other GO term.

Our semi-automated methodology can be readily applied to find GO terms with other properties, for example, inflammation-related GO terms or GO terms involving transcription. If the property is clearly defined and careful thought is given to establishing curation rules and choosing gold standard terms, it is straightforward with our methodology to find the rest of the terms with such a property.

### Curation

There are many issues and difficulties in the curation process. The first problem is to define the scope of lipid- relatedness, and different researchers may not agree with each other on this because of their different background, field of expertise, experiences and so on. We follow the definition of lipids from LIPID MAPS (14), as mentioned before in the KW feature generation section and curation section. LIPID MAPS gives eight main categories of lipids as well as sub-categories, derivatives of standard lipid compounds, etc. We mostly follow this system in our project except that we do not consider polyketides as lipid related, as mentioned in our curation rules.

Second, sometimes the term does not appear in a neat form like ‘metabolism involving X’, but in an implicit form, from which alone nothing can be said about whether the term is lipid related, and details about the process have to be known before decisions can be made. For instance, the BP term GO:0034238 has a term name ‘macrophage fusion’, containing no lipid-related KW. During this process, there is membrane fusion (curation rule 8) of macrophage with other cells into a multinucleated cell, we thus consider this term as lipid related. Curation rules 8, 9 and 10 are especially designed to identify lipid-related GO terms in implicit form and detailed knowledge for such kinds of term is often required of the curator. If the term in question is new to the curator, a considerable amount of time and effort is needed for him or her to gather information to make a decision, especially for BP terms, each consists of a series of events accomplished by one or more ordered assemblies of MFs. Furthermore, even with our set of rules, some terms still needed to be decided on a case-by-case basis. We nonetheless have tried our best to generalize our rules not only to make it clear to the end users but also to make the curation as consistent as possible, even by a newly trained curator.

With more experiences accumulated, we constantly refine and expand the existing set of curation rules to make it as consistent and informative as possible. We used to consider the CC term GO:0044425 ‘membrane part’ as lipid-related in our original gold standard. Many of its descendant terms are protein complexes embedded in membrane, which we now consider as not good enough to be qualified as lipid related unless additional criteria are satisfied: they are either intrinsic to membrane or involved in metabolism of lipids or lipid complexes, membrane folding and vesicle fusion (curation rule 12). For example, the term GO:0034702 ‘ion channel complex’, whose main function is allowing selective ion transport down its electrochemical gradient, is no longer considered as lipid related because it fails the criteria above. On the other hand, the term GO:0046696 ‘lipopolysaccharide receptor complex’ remains as CC+, as this complex involves lipid metabolism. By the inheritance constraint, we also flip the class label of the ancestor term GO:0044425 ‘membrane part’ from CC+ to CC− because there are now non–lipid-related terms among its descendants.

Moreover, terms in different versions of GO evolve. For example, there are changes in the term definition or changes in relationship to other terms. We thus change our curation rules to account for that. For example, the term definition for GO:0046459 ‘short-chain fatty acid metabolic process’ is changed from chemical reactions and pathways involving fatty acids with a chain length of <8 carbons to with a chain length of <6 carbons. Moreover, this term has a new term GO:0019541 ‘propionate metabolic process’ added as its child, which is not lipid related in the traditional sense. Therefore, we now set the class labels of both child and parent terms (GO:0019541 and GO:0046459) as BP−, and we generalized this situation to make rule 1.

## Conclusion

Searching lipid-related GO terms comprehensively across all three sub-ontologies of GO is a nontrivial problem, requiring a considerable amount of time and effort. Our methodology tackles this problem using a combination of computational and manual curation. Starting with a list of initial gold-standard terms, we expand our list of lipid-related GO terms incrementally. Each step we calculate feature scores for GO terms based on KWs and co-annotation of gene products, build a classifier and make a prediction followed by manual curation. During the process, inheritance constraint is enforced to ensure the integrity of classification in the GO structure. This incremental expansion methodology is able to extract lipid-related terms with high confidence without too much manual curation. Though not completely exhaustive, it is estimated that a great majority of lipid-related BP terms have been covered. Our semi-automated methodology can be used to find GO terms with other properties, not just lipid-relatedness.

## Funding

This work was supported in part by a Singapore National Research Foundation CRP grant (NRF-G-CRP-2007-04-082(d)) and a Singapore Ministry of Education tier-2 grant (MOE2012-T2-1-061). Funding for open access charge: MOE2012-T2-1-061.

*Conflict of interest*: None declared.

## Supplementary Material

Supplementary Data

## References

[1] Ashburner M. Ball C.A. Blake J.A. (2000) Gene Ontology: tool for the unification of biology. Nat. Genet., 25, 25–29 10802651 10.1038/75556 PMC3037419

[2] Gene Ontology Consortium. (2010) The Gene Ontology in 2010: extensions and refinements. Nucleic Acids Res., 38 (Suppl 1), D331–D335 19920128 10.1093/nar/gkp1018 PMC2808930

[3] Pesquita C. (2009) Semantic similarity in biomedical ontologies. *PLoS Comput. Biol*., 5, e1000443 19649320 10.1371/journal.pcbi.1000443 PMC2712090

[4] Resnik P. (1999) Semantic similarity in a taxonomy: an information-based measure and its application to problems of ambiguity in natural language. J. Artif. Intell. Res., 11, 95–130

[5] Lord P.W. Stevens R.D. Brass A. Goble C.A. (2003) Investigating semantic similarity measures across the Gene Ontology: the relationship between sequence and annotation. Bioinformatics, 19, 1275–1283 12835272 10.1093/bioinformatics/btg153

[6] Jiang J.J. Conrath D.W. (1997) Semantic similarity based on corpus statistics and lexical taxonomy. In: Proceedings of the 10th International Conference on Research on Computational Linguistics, Taipei, Taiwan, The Association for Computational Linguistics and Chinese Language Processing, Nankang, Taipei, Taiwan, pp 19–33

[7] Lin D. (1998) An information-theoretic definition of similarity. In: Proceedings of the Fifteenth International Conference on Machine Learning, Madison, Wisconsin, USA, Morgan Kaufmann Publishers Inc., San Francisco, CA, USA, pp. 296–304

[8] Schlicker A. Domingues F.S. Rahnenführer J. Lengauer T. (2006) A new measure for functional similarity of gene products based on Gene Ontology. BMC Bioinformatics, 7, 302 16776819 10.1186/1471-2105-7-302 PMC1559652

[9] Pekar V. Staab S. (2002) Taxonomy learning: factoring the structure of a taxonomy into a semantic classification decision. In: Proceedings of the 19th International Conference on Computational Linguistics, Taipei, Taiwan, Vol. 1, Association for Computational Linguistics, Stroudsburg, PA, USA, pp. 1–7

[10] Wang J.Z. Du Z. Payattakool R. (2007) A new method to measure the semantic similarity of GO terms. Bioinformatics, 23, 1274–1281 17344234 10.1093/bioinformatics/btm087

[11] Jin B. Lu X. (2010) Identifying informative subsets of the Gene Ontology with information bottleneck methods. Bioinformatics, 26, 2445–2451 20702400 10.1093/bioinformatics/btq449 PMC2944202

[12] Riensche R.M. Baddeley B.L. Sanfilippo A.P. (2007) XOA: Web-enabled cross-ontological analytics. Services, 2007 IEEE Congress, 99–105

[13] Wenk M.R. (2005) The emerging field of lipidomics. Nat. Rev. Drug Discov., 4, 594–610 16052242 10.1038/nrd1776

[14] Goh W.W. Fan M. Low H.S. (2013) Enhancing the utility of Proteomics Sig-nature Profiling (PSP) with Pathway Derived Subnets (PDSs), performance analysis and specialized ontologies, BMC Genomics, 14, 35 23324392 10.1186/1471-2164-14-35 PMC3636053

[15] Fan M. Low H.S. Zhou H. (2014) LipidGO: database for lipid-related GO terms and applications. Bioinformatics, 30, 1043–1044 24297518 10.1093/bioinformatics/btt689

[16] Fahy E. Subramaniam S. Murphy R. (2009) Update of the LIPID MAPS comprehensive classification system for lipids. J. Lipid Res.*,* 50, S9–S14 19098281 10.1194/jlr.R800095-JLR200 PMC2674711

[17] Hill D.P. Adams N. Bada M. (2013) Dovetailing biology and chemistry: integrating the Gene Ontology with the ChEBI chemical ontology. BMC Genomics, 14, 513 23895341 10.1186/1471-2164-14-513 PMC3733925

