# Supplementary material for: A semi-automated methodology for finding lipid-related GO terms
Source: Database (Oxford). 2014 Sep 10;2014:bau089. doi: 10.1093/database/bau089 (PMC4160098; doi:10.1093/database/bau089)
Supplement: Supplementary Data [file supp_2014_bau089_index.html]

A semi-automated methodology for finding lipid-related GO terms — Supplementary Data 

# A semi-automated methodology for finding lipid-related GO terms

## Supplementary Data

files

**Files in this Data Supplement:**

- Supplementary Data - txt file
- Supplementary Data - txt file
- Supplementary Data - txt file
